# Metabolic Dysfunctions, Dysregulation of the Autonomic Nervous System, and Echocardiographic Parameters in Borderline Personality Disorder: A Narrative Review

**DOI:** 10.3390/ijms252212286

**Published:** 2024-11-15

**Authors:** Paola Bozzatello, Giacomo Marin, Giulio Gabriele, Claudio Brasso, Paola Rocca, Silvio Bellino

**Affiliations:** Department of Neurosciences, University of Turin, Via Cherasco 15, 10126 Turin, Italy; giacomo.marin@unito.it (G.M.); giulio.gabriele@unito.it (G.G.); claudio.brasso@unito.it (C.B.); paola.rocca@unito.it (P.R.); silvio.bellino@unito.it (S.B.)

**Keywords:** borderline personality disorder (BPD), allostatic load (AL), cardiovascular risk, oxidative stress, echocardiographic parameters, autonomic alterations

## Abstract

Borderline personality disorder (BPD) is a complex psychiatric disorder characterized by an unstable sense of self and identity, emotional dysregulation, impulsivity, and disturbed interpersonal relationships. This narrative review examines the interplay between dysregulation of the autonomic nervous system, metabolic changes, and cardiovascular risk in BPD. Altered heart rate variability (HRV), reflecting the dysregulation of the autonomic nervous system, is associated with some BPD core symptoms, such as emotional instability and impulsivity. Dysregulation of the hypothalamic–pituitary–adrenal (HPA) axis, often stemming from early trauma, contributes to chronic inflammation and elevated allostatic load, which further increases cardiovascular risk. Metabolic dysfunctions in BPD, such as elevated body mass index (BMI), high blood pressure, and inflammatory markers like C-reactive protein (CRP), exacerbate these risks. Speckle-tracking echocardiography, particularly global longitudinal strain (GLS) and biomarkers such as homocysteine and epicardial fat, could be considered early predictors of cardiovascular events in individuals with BPD. Chronic stress, inflammation, and maladaptive stress responses further heighten cardiovascular vulnerability, potentially accelerating biological aging and cognitive decline. A literature search covering the period from 2014 to 2024 on PubMed identified 189 studies on this topic, of which 37 articles were deemed eligible for this review. These included cross-sectional, longitudinal, case–control, randomised controlled trials (RCTs), reviews, and meta-analysis designs, with sample sizes ranging from 14 to 5969 participants. The main limitations were that only one database was searched, the time of publications was limited, non-English manuscripts were excluded, and the quality of each paper was not commented on. This narrative review aims to provide an overview of recent evidence obtained on this topic, pointing out a direction for future research.

## 1. Introduction

The current definition of personality in psychiatry encompasses a complex and nuanced set of profound psychological traits that are largely unconscious, fundamentally stable, and that manifest in all areas of an individual’s mental life. This approach to interacting with reality, responding to events, and navigating one’s existence necessitates strong adaptive skills to meet the demands of the social environment. When these adaptive skills falter, the individual may develop a rigid and inflexible perception of reality, along with fixed thought patterns and behaviors, resulting in significant subjective distress and impaired functioning. This context defines personality disorders, including a group of severe and debilitating psychiatric conditions characterized by chronicity and a need for effective treatment [1]. Borderline personality disorder (BPD) is a severe personality disorder, with a prevalence in the general population estimated to be between 0.7% and 5.8% [2,3]. In clinical settings, this rate increases significantly, with BPD affecting up to 10% of psychiatric outpatients and 15 to 20% of inpatients [4,5,6]. Patients with BPD typically experience abrupt and frequent mood swings, a fragmented self-image, instability in interpersonal relationships, significant dyscontrol of impulses, and difficulty in understanding their own and others’ emotional and mental states [4]. Individuals with BPD may find themselves experiencing contrasting emotions simultaneously, resulting in confusion, anxiety, and distress. Patients often respond to environmental stressors through impulsive actions, reflecting reduced frustration tolerance and a poor ability to evaluate the consequences of their behaviors. A lack of impulse control is expressed by various risk-taking behaviors, such as the following: violent confrontations, substance misuse, binge drinking, high-risk sexual activities, gambling, and compulsive spending. Constant instability of these subjects is also shown in interpersonal interactions. Relationships tend to be tumultuous and deeply engaging, yet characterized by acute anxiety regarding abandonment, profound instability, and chaos, leading to swift shifts from idealizing others to devaluing them [4,7].

Difficulties in reflecting on one’s experiences, moods, and emotional connections, combined with the individual’s heightened sensitivity to stressors and emotional pressures, can lead to significant overall functional impairment, resulting in the loss of social roles, isolation, and marginalization [8]. The typical manifestations of BPD often overlap with symptoms of other psychiatric conditions, such as mood disorders, phobias, panic disorder, bulimia, and substance abuse.

In BPD, early exposure to psychosocial traumatic factors, combined with inadequate coping mechanisms, can often lead to chronic stress, which may promote the development of cardiovascular disease (CVD) [9]. Damage due to chronic stress can be mediated by oxidative factors, dysregulation of the hypothalamic–pituitary–adrenal (HPA) axis, and, consequently, modifications of metabolic parameters related to cardiovascular risk [10]. Low-grade chronic inflammation (elevated C-reactive protein levels) is registered in BPD patients, especially in subjects who have a history of traumatic events [11]. Chronic inflammation can be encapsulated within the concept of allostatic load. Allostatic load is an objective marker of a maladaptive stress response that includes several parameters (systolic and diastolic blood pressure, BP; waist-to-hip ratio; body mass index, BMI; heart rate, HR; white blood cell count; total cholesterol; high-density lipoproteins, HDL; triglycerides; hemoglobin A1c, HbA1c; albumin; creatinine; C-reactive protein, CPR). An aberrant somatic response to stress could contribute to metabolic syndrome (increased abdominal fat, insulin dysregulation, dyslipidemia, hypertension) with a consequent increase in cardiovascular risk (stroke, infarction) [12,13].

In order to identify possible predictive markers that allow us to prevent cerebral and cardiovascular accidents early, some echocardiographic parameters have been considered. In particular, speckle-tracking echocardiography specifically evaluates the global longitudinal strain (GLS). Other assessable parameters that could be considered are the blood value of homocysteine and the presence of ectopic fat (intra-abdominal and, in particular, epicardial) [9,10,14].

The increased cardiovascular risk is not only related to the dysregulated stress response, but also to an increase in modifiable lifestyle risk factors. In particular, there is evidence of greater smoking habits among BPD patients, a dysregulated and less attentive diet, and more difficulty adhering to internist therapies compared to the general population. Additionally, several psychiatric drugs used may cause or worsen metabolic syndrome [15].

Furthermore, traumatic events in BPD patients during early childhood seem to contribute to the dysregulation of the autonomic nervous system (ANS) [16]. A growing body of research suggests that ANS abnormalities play a pivotal role in the pathophysiology of BPD. ANS dysregulation is often indexed by heart rate variability (HRV), which reflects the balance between sympathetic and parasympathetic activities. Several studies have indicated that individuals with BPD exhibit altered HRV and autonomic functioning, suggesting that these physiological markers may be linked to emotional dysregulation and impulsivity [17]. In summary, traumatic childhood experiences in predisposed patients may lead to dysregulation of the autonomic nervous system, which in turn results in an altered stress response with an increased allostatic load, worsening of the metabolic syndrome parameters, dysregulation of the HPA axis, and low-grade inflammation. The hyperresponsiveness of the HPA axis might be due to the enhanced central drive to pituitary ACTH release, and it is associated with chronic childhood abuse [18]. Furthermore, structural dissimilarities of the brain in people with borderline personality disorder are highlighted, such as smaller amygdala, hippocampus, anterior cingulate, and orbitofrontal cortex volumes [19,20].

This narrative review aims to provide an updated overview of the existing evidence regarding metabolic aspects, echocardiographic findings, and abnormalities of the autonomic nervous system in BPD. This approach facilitates a more holistic understanding of BPD patients, incorporating biological factors into their evaluation, treatment, monitoring, and care planning.

## 2. Results

**Figure 1:**
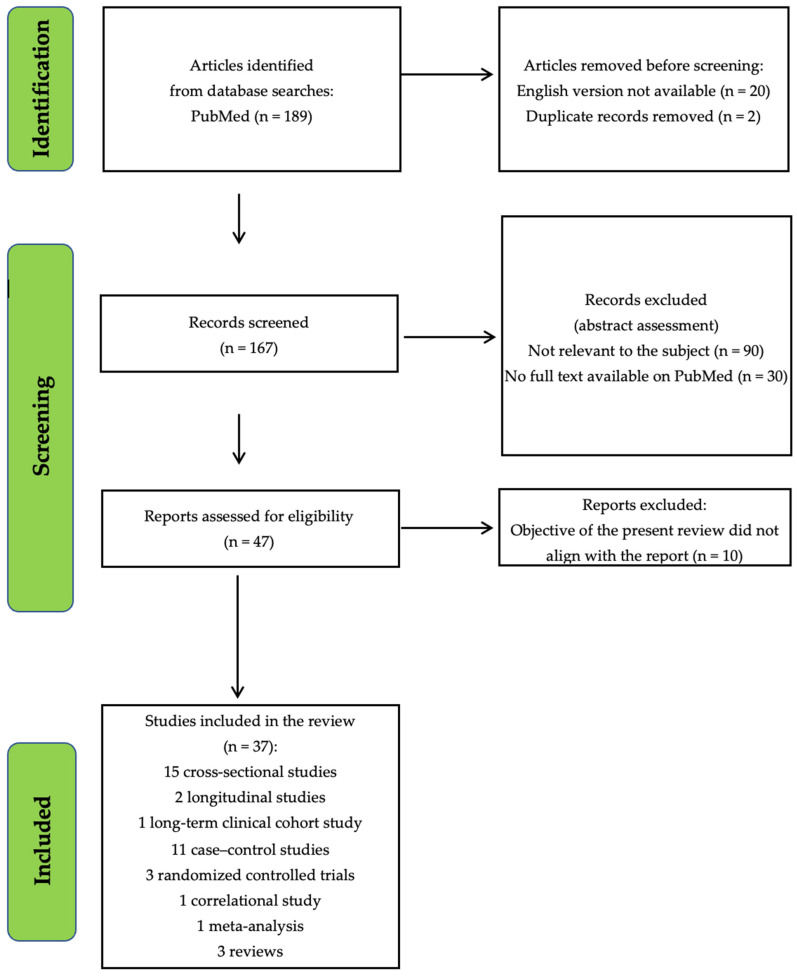
Illustration of the records obtained from PubMed, along with the subsequent screening process for the studies.

Records from PubMed and study screening are displayed in the following flowchart (Figure 1).

## 3. Discussion

### 3.1. Dysregulation of the Autonomic Nervous System in BPD

A growing body of research suggests that dysregulation of the autonomic nervous system plays a pivotal role in BPD pathophysiology. This dysregulation is often indexed by heart rate variability (HRV), which reflects the balance between sympathetic and parasympathetic activities. Several studies have indicated that individuals with BPD exhibit altered HRV and autonomic functioning, suggesting that these physiological markers may be linked to emotional dysregulation and lability, negative affectivity, disinhibition (impulsivity and risk-taking), reduced adaptability and behavioral flexibility, alexithymia, and dissociation, which are typical symptoms of the disorder [17,21,22,23]. While there is a consensus on the presence of ANS dysregulation in BPD, variations in findings and methodologies across studies warrant a comprehensive synthesis of the existing literature. A meta-analysis that considered five studies with a total sample of 128 patients with borderline personality disorder highlighted a consistent relationship between core dimensions of BPD—such as negative affectivity, emotional lability, anxiety, depression, and disinhibition—and a lower resting-state vagal tone, measured through vagally-mediated heart rate variability (vmHRV). This indicates that reduced vmHRV may reflect a common psychophysiological mechanism contributing to the difficulties in emotion regulation and impulsivity observed in individuals with BPD [17]. In addition, some studies hypothesized that HRV serves as a non-invasive measure of ANS regulation [2,24,25]. Some investigators reported decreased HRV in BPD, suggesting increased sympathetic and decreased parasympathetic activity [26,27], while others provide conflicting evidence [25,28]. In a longitudinal study, tracking 17 female adolescents with BPD over one year, changes in resting vmHRV and heart rate were associated with fluctuations in BPD symptoms, suggesting vmHRV as a biomarker for emotional regulation capacity in BPD [29]. Other authors discussing this finding suggested that lower vmHRV has been linked to both physical and mental health outcomes, reflecting low emotional regulation and behavioral flexibility, traits that are often compromised in BPD patients [23].

The connection between HRV and social behaviors in BPD has also been studied. Maiß et al. (2021), in a case–control study that recruited 42 female patients with BPD, confirmed that heart rate variability (HRV) is overall lower in BPD patients compared to controls [30]. They also associated this reduction in HRV with social behavior, specifically interpersonal avoidance behaviors. In addition, a case–control study by Eddie et al., involving 22 patients with BPD, investigated psychophysiological responses during exposure to emotional stimuli. The study revealed that BPD patients, particularly those with more severe symptoms, exhibited higher heart rates, indicative of heightened disruptions in sympathetic and parasympathetic activity [31]. These data highlighted the complex interactions between autonomic dysregulation and emotional challenges in BPD.

Research focusing on adolescent populations has identified significant associations between reduced resting-state heart rate variability (HRV) and the severity of borderline personality disorder (BPD) symptoms. In a cross-sectional analysis, Weise et al. studied a group of 43 adolescent patients with BPD (ages 12–18) and found that greater symptom severity correlated with reduced HRV and increased heart rate, linking autonomic nervous system (ANS) dysfunction to the overall severity of BPD symptoms. The authors also highlighted the existence of a significant relationship between HRV and current levels of functioning, suggesting that poorer psychosocial functioning was associated with lower HRV [32].

Autonomic dysregulation in BPD patients was also found in terms of lower interoceptive awareness than in healthy controls [33]. One study stated that BPD patients (n = 20) exhibited altered cortical processing of interoception, with greater sympathetic activity and heightened heartbeat-evoked potentials (HEP) compared to controls (n = 20). So, difficulties in emotional awareness and regulation may stem from both autonomic dysregulation and impaired interoceptive processing [22]. Another investigation reported that BPD patients (n = 29) displayed a reduced vagal tone and overall autonomic cardiac modulation, alongside a similar sympathetic modulation compared to controls, indicating impaired parasympathetic responses linked to emotional regulation difficulties [34].

The emotional reactivity patterns observed in patients with borderline personality disorder (BPD) suggest a lack of adaptive responses to emotional challenges. For example, some authors have shown that BPD patients do not exhibit typical bradycardia responses to unpleasant stimuli, indicating a predominant reliance on fight-or-flight mechanisms instead of flexible emotional responses [21]. These findings challenge the notion that emotional hyperresponsivity is the key feature of BPD. They emphasize a more complex perspective on emotional dysregulation, where reduced adaptability and a lack of behavioral flexibility play central roles.

Another study found that the lower heart rate response to stress in adolescents with BPD contradicts the idea of hyperresponsivity in this disorder [35]. In their cross-sectional study, Kaess et al. investigated dual-task performance in 30 female adolescents with BPD compared to 34 age-matched controls under both stress and non-stress conditions. The healthy controls showed an increase in heart rate after stress induction, while the BPD group did not exhibit any change. This reduced heart rate response to acute stress in adolescents with BPD challenges the theory that BPD patients exhibit stronger stress reactivity. BPD may involve both hyperresponsiveness and hyporesponsiveness to stress, influenced by the nature of the stressor. Findings indicate that decreased parasympathetic activity in resting BPD patients, suggesting a baseline of increased arousal, may lead to blunted reactions to moderate stressors.

The impact of trauma on autonomic functioning is further supported by studies showing that individuals with BPD and comorbid post-traumatic stress disorder (PTSD) exhibit significantly lower HRV [16]. HRV aberrations have been posited as potentially linked to early-life maltreatment and psychological factors, rather than being strictly tied to the BPD diagnosis itself [25]. Interestingly, higher self-reported dissociation predicted increased high-frequency HRV (HF-HRV) during emotion regulation tasks, highlighting the influence of dissociative mechanisms on autonomic regulation in BPD, particularly in the context of trauma.

Some investigations were conducted to better understand the interplay between autonomic response, resting-state HRV, and childhood trauma, including attachment insecurity in BPD. A cross-sectional study involving 40 participants—20 with BPD and 20 healthy controls—observed that exposure to adverse childhood experiences (ACEs) was associated with distinct patterns of autonomic nervous system (ANS) activation during emotional regulation [36]. Participants underwent a cognitive reappraisal task while undergoing functional magnetic resonance imaging (fMRI) with simultaneous electrocardiogram (ECG) acquisition. ACEs affect neurophysiological mechanisms in BPD by altering the top-down control of autonomic responses, particularly involving the anterior cingulate cortex and medial superior frontal gyrus. This alteration results in distinct brain–autonomic coupling during emotion regulation. Individuals with BPD exhibit a unique activation pattern in these prefrontal regions, reflecting an effortful yet altered regulation of parasympathetic activity during emotional processing. These results are consistent with those of another investigation, showing that higher levels of childhood traumas significantly predict reduced HRV [37]. Back et al. conducted a randomized controlled trial involving 113 women, of whom 53 had BPD and 60 were healthy controls, to investigate predictors of reduced resting-state heart rate variability (HRV) in BPD. The study focused on the interaction between childhood trauma and adult attachment insecurity. The findings revealed significantly reduced HRV in women with BPD, which was associated with both childhood trauma and attachment insecurity. Specifically, at high levels of attachment insecurity, childhood trauma was found to predict lower HRV. This underscores the importance of early adverse experiences in influencing autonomic regulation and emotional dysregulation in BPD.

In a neuroimaging study, Villarreal et al. highlighted that autonomic alterations are not exclusive to BPD, but are also present in other psychiatric disorders, such as major depressive disorder (MDD). The study examined the activation of the central autonomic network (CAN) during stress in patients with BPD (n = 24) and MDD (n = 24), as well as healthy controls (n = 25). It found that both patient groups exhibited less intense CAN activation compared to healthy participants, along with increased sympathetic and decreased parasympathetic activation. These findings suggest that autonomic dysregulation affects both BPD and MDD patients, and may be more related to the impact of emotional dysregulation on autonomic functioning than to the specific diagnosis [38].

In summary, the available evidence on autonomic nervous system dysregulation in BPD reveals a complex interplay between physiological and psychological factors. While there is a consensus on the existence of reduced HRV and altered autonomic functioning in BPD, the specific mechanisms and implications of these changes remain an area of active research. Studies consistently demonstrate that lower vagal tone and sympathetic predominance are linked to emotional dysregulation and impulsivity in BPD. Additionally, the impact of childhood trauma and attachment insecurity highlights the importance of early experiences in shaping autonomic responses. It also needs to be established whether these alterations are specific to borderline personality disorder or common to other disorders that share similar symptomatology.

### 3.2. Metabolic Dysfunctions and Cardiovascular Risk in BPD

The literature regarding the association between the diagnosis of BPD and an increased cardiovascular risk is not very extensive. Nevertheless, some studies with large samples are available [15,39,40]. Most of these studies have identified a possible association between BPD and elevated biomarkers that are known to be linked to increased cardiovascular risk, such as high-density lipoproteins (HDL), low-density lipoproteins (LDL), hemoglobin A1c (HbA1c), and C-reactive protein (CRP), as well as measured values like systolic and diastolic blood pressure, waist-to-hip ratio, heart rate, and body mass index (BMI) [9,12,13,15,39,40,41,42,43]. Values of these parameters, which are consequently associated with an increased risk of cardiovascular events, were generally elevated in patients with BPD.

Of particular note is the study by Baptista et al. (2023), which, in a sample of more than 34,000 subjects, of which 892 were affected by BPD, demonstrated a statistically significant increase in metabolic factors implicated in cardiovascular risk. Additionally, this study confirmed an increased prevalence of childhood traumatic events in patients with BPD, raising the question of whether the heightened cardiovascular risk is related to the diagnosis of BPD itself, or whether it is due to the presence of early traumatic events [15]. Studies by Cheney et al. and Kern et al. reported an increased BMI in 104 patients diagnosed with BPD in comparison with a control group of healthy controls [40,42]. Cheney et al. observed an average BMI of 28.57 kg/m^2^ in the BPD group, compared to 22.40 kg/m^2^ in the control group. This difference may be related to a less regulated lifestyle, including poorer dietary choices and greater impulsivity, which also extends to eating behaviors. Moreover, the Cheney et al. study found a significant association between a history of early traumatic events and increased BMI. Mediation analysis revealed that the relationship between sexual assault and BMI was fully mediated by BPD and depression [40]. The authors stressed the importance of intervention development, and that interventions tailored for certain populations of women incorporate trauma and psychological-based approaches, along with behavioral health approaches, to weight management.

Abrahamian et al. (2023) further highlight the issue of metabolic health by referencing findings from a cross-sectional study by Kahl et al. (2012), which revealed a significantly higher age-standardized prevalence of metabolic syndrome in patients with borderline personality disorder, twice as high as in the healthy control group with no psychiatric disorders (23.3% vs. 10.6%). The authors hypothesized that this data could be due to poor therapy adherence, low physical activity, and dysregulation of the hypothalamic–pituitary–adrenal (HPA) axis (which will be described later in the review) [44,45].

In addition to obesity, increased blood pressure—particularly diastolic blood pressure—was identified as another parameter linked to increased cardiovascular risk in BPD patients. A rise in blood pressure seems to be associated with heightened responsiveness to external stressors in this clinical population [34,43]. These findings were supported by a systematic review by Roininen et al. (2019), which suggested that patients with BPD could potentially benefit from cardiovascular disease prevention strategies, including hypertension management [41]. There is a general consensus in retaining that the increased cardiovascular risk in BPD patients is linked to a maladaptive physiological response to external or internal stressors, a concept theorized within the framework of allostatic load (AL). Allostatic load is an objective marker of a maladaptive stress response. The concept of AL suggests that chronic maladaptive responses to stress result in a somatic burden, leading to accelerated deterioration of the body beyond what would be expected for a given chronological age [12,13]. In a study by Otto et al. [12], it was observed that allostatic load positively correlated with BPD, aggression, and chronic stress. Brune and Otto [12] have provided a theoretical explanation for the elevated physical and biochemical values (systolic and diastolic blood pressure, waist-to-hip ratio, BMI, heart rate, white blood cell count, total cholesterol, LDL, triglycerides, HbA1c, albumin, creatinine, and CRP) observed in BPD patients. Drawing on Life History Theory (LHT), they proposed that individuals who anticipate a future lack of resources may adopt a ‘faster’ life history strategy, prioritizing reproductive activity over somatic maintenance and tissue repair. According to this theory, early social stressors, such as abuse or neglect, may biologically ‘prepare’ individuals to face future threats, potentially contributing to the increased cardiovascular risk observed in BPD patients. This higher AL could represent the somatic consequence of a ‘fast’ life history strategy due to decreased investment in bodily maintenance and repair [12].

Chen et al. (2017), in a nationwide longitudinal study involving 5969 patients with BPD aged over 18, demonstrated that patients with BPD had a significantly higher incidence of developing any type of stroke, ischemic stroke, and hemorrhagic stroke. They also experienced an earlier onset of any stroke and ischemic stroke compared to controls. The underlying mechanism, as highlighted by the authors, remains unclear. The authors propose a biopsychosocial hypothesis to explain the relationship between BPD and stroke. From a biological perspective, patients with BPD exhibit dysregulation of the HPA axis and altered levels of stress hormones, such as cortisol, which are associated with dissociative, borderline, and depressive symptoms. Additionally, elevated levels of pro-inflammatory cytokines, including tumor necrosis factor α (TNF-α) and interleukin 6 (IL-6), in these patients are linked to vascular damage and an increased risk of cardiovascular and cerebrovascular diseases. Furthermore, BPD is associated with higher concentrations of vascular endothelial growth factors (VEGF) and fibroblast growth factors (FGF-2), both of which contribute to endothelial dysfunction and vasculopathy. Several studies have suggested that BPD could be considered a significant risk factor for atherosclerosis and ischemic stroke. From a psychosocial standpoint, individuals with BPD are particularly vulnerable to psychosocial trauma and chronic stress. Chronic stress and traumatic experiences can elevate stroke-related risk factors, including hypertension, dyslipidemia, and diabetes. Moreover, the dysregulation of the HPA axis due to stress further heightens the risk of stroke. Finally, endothelial dysfunction, exacerbated by stress and depressive symptoms, increases susceptibility to vascular diseases [45].

Beyond metabolic alterations, other predictors of cardiovascular risk have also been investigated. A study by Richter et al. evaluated the excess presence of epicardial adipose tissue (EAT) and intra-abdominal adipose tissue (IAT), as assessed by nuclear magnetic resonance imaging in a sample of 76 patients (28 with BPD and comorbid diabetes mellitus (DM), 22 with DM alone, and 26 controls). Epidemiological studies suggest that IAT and EAT are independent markers of cardiovascular and metabolic morbidity and mortality. Emerging evidence also indicated that ectopic fat deposition, including EAT, contributes to increased atherosclerosis and cardiometabolic risk. In this study, an increase in EAT was observed in BPD patients for the first time, underscoring the heightened cardiovascular risk in this psychiatric population [10]. The deposition of ectopic fat may be mediated by chronic hyperactivation of the hypothalamic–pituitary–adrenal (HPA) axis, resulting in low-grade inflammation, as indicated by elevated CRP levels [11,46].

Another attempt to identify cardiovascular risk markers in BPD patients focused on homocysteine (Hcy). Elevated plasma Hcy levels have been identified as a risk factor for vascular disease, independent of other risk factors such as hypercholesterolemia, hypertension, or smoking. There is mounting evidence that suggests the associations between elevated Hcy levels and coronary heart disease, cardiovascular disease, and cerebrovascular disease. Furthermore, Hcy probably plays a role in endothelial dysfunction. In a study by Kern et al., young female BPD patients were found to have higher Hcy levels compared to healthy controls, although the clinical significance of this finding requires further investigation and replication [42]. Similarly, Wang et al. (2024) found that the presence of both bipolar disorder and BPD in a cohort of 60 patients was associated with elevated serum Hcy and high-sensitivity CRP levels [14]. These findings reinforce the hypothesis that BPD is associated with an elevated risk of poor somatic health. A more speculative conclusion presented in the study suggests that Hcy could potentially serve as an indicator for monitoring cardiovascular health in this patient group.

Among the diagnostic procedures evaluating the increase in cardiovascular risk, recent investigations have been focused on a specific echocardiographic parameter assessable via speckle-tracking echocardiography (STE). This parameter is the ventricular global longitudinal strain (GLS). A reduction of GLS has been associated with an increased risk of cardiovascular mortality [13]. In an interesting study by Aweimar et al., involving 100 subjects, the diagnosis of BPD was found to be statistically associated with reduced GLS. However, the study raised the question of whether this association is due to the diagnosis itself or confounding factors such as obesity and smoking, both of which are more prevalent in BPD patients [9]. A similar investigation by Engelman et al. (2022), with a similar sample, observed a significant association between BPD, increased allostatic load, reduced GLS, and childhood traumatic events. In contrast to Aweimar’s study, Engelman et al. found no association between smoking and reduced GLS, opening up the possibility that altered lifestyles are not so related to the reduction in GLS [13].

In summary, there is a significant association between borderline personality disorder (BPD) and increased cardiovascular risk. Patients with BPD often exhibit elevated biomarkers, such as cholesterol, HbA1c, C-reactive protein, and heightened blood pressure, indicating an overall metabolic dysregulation. Factors such as obesity and a history of childhood trauma further contribute to this elevated risk, while an increased allostatic load suggests that maladaptive stress responses play a critical role. Additionally, individuals with BPD have a higher incidence of stroke, which is linked to both biological dysregulation and chronic stress. These findings underscore the urgent need for targeted strategies to improve cardiovascular health in this population.

### 3.3. Inflammation and HPA Axis Dysregulation in BPD

The role of inflammation in BPD is gaining attention, as emerging evidence suggests a complex interplay between physiological stress responses, inflammatory markers, and the psychopathology of this disorder [46,47]. In BPD patients, a significant dysregulation of the hypothalamic–pituitary–adrenal (HPA) axis was observed, which was characterized by lower baseline cortisol levels and a blunted response to stress compared to healthy controls. In addition, these patients often show heightened skin conductance levels (SCL), indicating an autonomic nervous system imbalance, despite reduced heart rate responses. Such dysregulation is frequently linked to early-life trauma, which may contribute to a maladaptive physiological response to chronic stress and promote inflammatory processes [46].

Moreover, the relationship between trauma and HPA axis dysfunction can lead to low-grade inflammation, as evidenced by elevated levels of C-reactive protein (CRP). The inflammatory response is particularly pronounced in BPD patients with co-occurring post-traumatic stress disorder (PTSD); thus, we can hypothesize that traumatic experiences play a crucial role in exacerbating inflammatory processes [11]

The long-term effects of chronic stress and inflammation may also manifest in accelerated biological aging. Research indicates that women with BPD, particularly those with a history of suicidal behaviors, may experience epigenetic age acceleration (EAA), implying that the persistent psychological and physiological stress associated with BPD can hasten the aging process [47]. The association of BPD with elevated levels of homocysteine (Hcy) and high-sensitivity C-reactive protein (hs-CRP) suggests that these inflammatory markers may contribute to cognitive impairment. This relationship highlights the potential for inflammation to exacerbate cognitive dysfunction, particularly in patients with both BPD and bipolar disorder [14]

In conclusion, these findings collectively underscore the role of chronic stress, trauma, and HPA axis dysregulation in promoting inflammation in BPD, which may contribute to accelerated aging and cognitive vulnerability. Further research is necessary to elucidate the intricate mechanisms linking inflammation to the psychopathology of BPD.

## 4. Methods

In September 2024, an electronic search was conducted on PubMed regarding metabolic aspects, echocardiographic parameters, and dysregulation of the autonomic nervous system in borderline personality disorder, using the following search string:

((((“Borderline Personality Disorder”[Majr] OR borderline-personality-disorder*[tiab]))) AND ((((“Waist-Hip Ratio”[Mesh] OR “Body Mass Index”[Mesh] OR “C-Reactive Protein”[Mesh] OR “Lipoproteins, HDL”[Mesh] OR “Glycated Hemoglobin”[Mesh] OR “Blood Pressure”[Mesh] OR “Hypertension”[Mesh] OR “Diabetes Mellitus”[Mesh] OR “Global Longitudinal Strain”[Mesh] OR “Ventricular Function, Left “[Mesh] OR “Echocardiography”[Mesh] OR “Cholesterol/administration and dosage”[Mesh] OR “Cholesterol/analysis”[Mesh] OR “Cholesterol/blood”[Mesh] OR “Heart Rate”[Mesh] OR “Leukocyte Count”[Mesh] OR “Triglycerides”[Mesh] OR “Creatinine”[Mesh] OR “Albumins”[Mesh] OR “Allostasis”[Mesh] OR allostas*[tiab] OR allostatic-load*[tiab] OR AL[tiab] OR albuminemia*[tiab] OR creatininemia*[tiab] OR trygliceride*[tiab] OR leukocyte-count*[tiab] OR leukocyte-number*[tiab] OR White-Blood-Cell-Count*[tiab] OR heart-rate*[tiab] OR cardiac-rate*[tiab] OR Heartbeat*[tiab] OR left-ventricular-myocardial-deformat*[tiab] OR Waist-hip-ratio*[tiab] OR body-mass-index[tiab] OR BMI[tiab] OR C-reactive-protein*[tiab] OR hs-CRP[tiab] OR HDL-lipoprotein*[tiab] OR High-Density-Lipoprotein*[tiab] OR Glycated-hemoglobin[tiab] OR Glycohemoglobin[tiab] OR HbAc1[tiab] OR Systolic-Pressure*[tiab] OR diastolic-pressure*[tiab] OR blood-pressure*[tiab] OR Hypertension OR Diabetes-Mellitus OR Global-longitudinal-strain[tiab] OR GLS[tiab] OR speckle-tracking-echocardiograph*[tiab]))))) AND (2014:3000/12/12[pdat]).

We considered publications published from January 2014 through September 2024. The inclusion and exclusion criteria for article selection were established before the literature search.

The included studies focus on metabolic dysfunction, autonomic nervous system dysregulation, and echocardiographic parameters in individuals with borderline personality disorder, involving participants aged 12 and older. Inclusion criteria encompassed original research articles, both observational and experimental studies, as well as reviews and meta-analyses that referred to recognized diagnostic criteria, specifically those outlined in the Diagnostic and Statistical Manual of Mental Disorders, from the fourth edition (DSM-IV) to the fifth edition text revision (DSM-5-TR). Eligible studies were required to include clinical assessments for BPD based on validated evaluation tools. Exclusion criteria are the following: studies outside of the relevant scope (e.g., those focused on pharmacological treatments, psychotherapeutic interventions, evaluation instrument validation, or social factors), specific article formats (e.g., comments and letters), and studies with overlapping data. Only studies published in English and with the full text available on PubMed were considered.

Studies were screened for eligibility by three authors independently (PB, GM, GG). Potentially eligible studies were identified based on titles and abstracts. The full texts of the potential target papers were then assessed for eligibility. Information on study design, sample size, inclusion and exclusion criteria, methods, and results was extracted independently by PB, GM, and GG. Disagreements were resolved in a consensus meeting with other reviewers (SB, CB, PR). The search algorithm resulted in a total of 189 articles, of which 167 were examined as potentially eligible studies. During the abstract screening, 90 articles were excluded due to their lack of relevance to the review topic, and 30 were excluded due to the unavailability of the full text. An additional 10 articles were excluded during the full text analysis because they did not align with the topic. Thirty-seven articles were retained for the final step.

We included the following types and numbers of publications: 15 cross-sectional studies, 2 longitudinal studies, 1 long-term clinical cohort study, 11 case–control studies, 3 randomized controlled trials, 1 correlational study, 1 meta-analysis, and 3 reviews 37 studies.

The number of participants in the studies ranged from 14 to 5969, with ages spanning from 12 to 65 years. No ethnicities were excluded; however, the majority of participants were Caucasian.

Information about the selected studies can be found in Table 1, Table 2 and Table 3.

## 5. Conclusions

In conclusion, the available evidence on the intricate relationships among the dysregulation of the autonomic nervous system, metabolic changes, and inflammatory processes in BPD suggests that autonomic dysregulation, as evidenced by reduced heart rate variability (HRV), is linked to emotional dysregulation and impulsivity, two core symptoms of BPD. Additionally, the presence of metabolic alterations, including elevated biomarkers and increased cardiovascular risk, indicates that individuals with BPD may face significant health challenges, stemming from both their psychological condition and early adverse experiences. Moreover, dysregulation of the hypothalamic–pituitary–adrenal (HPA) axis, often exacerbated by trauma, contributes to chronic inflammation, further complicating the clinical picture, and potentially accelerating biological aging. These interconnected factors emphasize the need for a comprehensive approach to treatment that addresses not only the psychological symptoms of BPD, but also the underlying physiological and metabolic health issues.

We emphasize that having standardized methods for data collection is important, particularly in the assessment of HRV, which is not always measured using consistent techniques across the numerous studies on the subject. Furthermore, except for a few investigations into metabolic impairment, most studies are characterized by small sample sizes. This highlights the potential usefulness of a meta-analysis to enhance the significance of the data obtained. Additionally, the studies are more representative of a female population, partially due to the more frequent seeking of medical help by female BPD patients. Most of the studies encountered are observational case–control studies, highlighting the need for further randomized experimental studies.

Further limitations arise from the fact that a narrative review should not be overly narrow (high precision), nor excessively broad (high return); however, it is typically broader in scope compared to systematic reviews. Consequently, relevant studies can be missed, and the reproducibility of the findings may be compromised. Additionally, the risk of bias and the methodological quality of the included studies are not formally assessed. The main other limitations were that only one database was searched, the time of publications was limited, and non-English manuscripts were excluded.

We believe it is important to continue searching for validated cardiovascular risk markers in BPD patients (e.g., GLS) to improve therapeutic and preventive strategies.

Future research should continue to explore these relationships, employing standardized methodologies to clarify the role of the ANS in BPD and its potential as a target for therapeutic interventions. Understanding the intricate connections in BPD patients between emotional regulation, ANS functioning, metabolic dysregulation, increased cardiovascular risk, and trauma history may enhance our ability to develop effective treatments for individuals with BPD.

## Figures and Tables

**Table 1:** Studies on dysregulation of the autonomic nervous system in BPD.

Authors	Study Design	States	Sample Characteristics	Outcomes
Meyer et al. [25]	case–control study	Germany	91 unmedicated female patients (18 with PTSD, 27 with current BPD, 23 subjects that did not fulfill BPD criteria, 23 healthy subjects)	The variance of HRV was higher in patients with BPD compared to those with PTSD.
Kaess et al. [35]	cross-sectional study	Germany	30 patients with BPD symptoms; 34 healthy controls (12–18 yo female)	Lower mean HR after stress induction in the BPD group.
Koenig et al. [17]	cross-sectional study	Germany	30 adolescents with BPD symptoms; 30 healthy controls (12–17 yo)	Resting-state HR and vmHRV in adolescents with NSSI were significantly correlated with BPD symptoms and their current level of functioning.
Koenig et al. [29]	longitudinal study	Germany	17 BPD female adolescents with NSSI	Changes in resting vmHRV and HR were associated with alterations in BPD symptoms.
Stoffels et al. [21]	case–control study	Netherlands	23 BPD patients	BPD patients did not exhibit bradycardia in response to unpleasant pictures, unlike healthy controls, regardless of their tendencies toward emotional avoidance.
Eddie et al. [31]	case–control study	New Jersey	22 BPD patients	Higher HR and SCR variability in individuals with BPD.
Krause-Utz et al. [16]	cross-sectional study	Netherlands	37 BPD patients; 20 BPD–PTSD patients; 27 healthy controls (18–55 yo)	Patients with BPD and PTSD exhibited significantly lower HF-HRV compared to the other groups.
Bortolla et al. [48]	cross-sectional study	Italy	14 BPD patients; 14 healthy controls (20–39 yo, female)	Constant hyperarousal state (lower RSA) observed in the BPD group.
Weise et al. [32]	cross-sectional study	Germany	43 patients with BPD symptoms (12–18 yo)	BPD symptom severity was associated with reduced resting-state HRV and increased heart rate.
Flasbeck et al. [22]	case–control study	Germany	20 BPD patients	BPD patients showed higher HEP amplitudes over frontal brain regions and increased sympathetic ANS activity.
Geiss et al. [34]	case–control study	Germany	29 BPD patients	BPD patients exhibited reduced vagal tone, decreased overall autonomic cardiac modulation, impaired baroreflex sensitivity, higher blood pressure, and shorter RR intervals.
Back et al. [37]	RCT	Germany	53 BPD patients/ 60 healthy controls (>18 yo, female)	Reduced HRV in women with BPD; no significant effect of oxytocin on mean HRV.
Maiß et al. [30]	case–control study	Germany	42 BPD patients (female)	Lower HRV in BPD patients.
Sigrist et al. [23]	long-term clinical cohort study (two years)	Germany	27 female adolescents with BPD symptoms (13–17 yo)	Reduced vmHRV in individuals with BPD.
Villarreal et al. [38]	case–control study	Argentina	24 BPD patients; 24 MDD patients; 25 healthy controls (18–65 yo)	CAN activation during stress was less intense in patients with BPD and MDD compared to healthy participants.
Wainsztein et al. [36]	cross-sectional study	Argentina	19 BPD patients; 20 MMD patients; 20 healthy controls (18–64 yo)	Individuals with BPD exhibit altered brain–autonomic coupling and unique prefrontal activation patterns during emotional regulation, reflecting an effortful yet impaired regulation of parasympathetic activity due to ACEs.
Weise et al. [49]	cross-sectional study	Germany	42 patients with BPD symptoms (12–18 yo)	Pre-treatment resting HRV predicted clinical improvement over time.
Krause-Utz et al. [50]	cross-sectional study	Netherlands	35 BPD patients; 18 BPD–PTSD patients; 28 healthy controls (18–55 yo, female)	Patients with BPD, particularly those with PTSD, demonstrated reduced HF-HRV, both at rest and during the EWMT.

vmHRV: vagally-mediated heart rate variability; BPD: borderline personality disorder; mRR: mean RR interval; HF: heart frequency; SCR: skin conductance reactivity; HEP: heartbeat-evoked potentials; PTSD: post-traumatic stress disorder; EWMT: emotional working memory task; RSA: respiratory sinus arrhythmia; ACEs: adverse childhood experiences; MDD: major depressive disorder; NSSI: non-suicidal self-injuries; CAN: central autonomic network.

**Table 2:** Studies on inflammation and HPA axis dysregulation in BPD.

Authors	Study Design	States	Sample Characteristics	Outcomes
Spitzer et al. [11]	cross-sectional study	Germany	12 PTSD patients (8 BPD–PTSD patients); 38 with no PTSD (27 BPD patients)	In BPD, when trauma is present (BPD–PTSD), dysfunction of the hypothalamic–pituitary–adrenal axis may contribute to low-grade inflammation, as indicated by elevated CRP levels.
Aleknaviciute et al. [46]	case–control study	Netherlands	26 BPD patients; 20 CPD patients; 35 healthy controls (female)	BPD patients showed distinct physiological patterns, as follows: lower baseline cortisol, blunted stress responses in cortisol and HR, but higher SCL, indicating autonomic imbalance. These attenuated responses were linked to HPA axis hyporeactivity, potentially due to early-life trauma.
Boström et al. [47]	RCT	Sweden	97 BPD females with prior history of two or more potentially lethal suicide attempts vs. 32 controls (18–50 yo)	Women with BPD and a recent history of suicide attempts exhibit EAA compared to healthy controls.
Wang et al. [14]	correlational study	China	60 BD–BPD patients (18–45 yo)	Elevated serum levels of Hcy and hs-CRP may regulate inflammatory responses, exacerbating cognitive impairment in patients with BD and BPD.

BPD: borderline personality disorder; CPD: cluster c personality disorders; HPA: hypothalamic–pituitary–adrenal axis; PTSD: post-traumatic stress disorder; HR: heart rate; Hcy: homocysteine; BD: bipolar disorder; hs-CRP: C-reactive protein; EAA: epigenetic age acceleration; SCL: skin conductance levels.

**Table 3:** Studies on metabolic dysfunctions and cardiovascular risk in BPD.

Authors	Study Design	States	Sample Characteristics	Outcomes
Cheney et al. [40]	cross-sectional study	United States	894 subjects (54 BPD patients) (18–52 yo)	Greater BMI was positively associated with BPD.
Grove et al. [43]	cross-sectional study	United States	143 subjects (23.2 medium age)	BPD features predicted elevated diastolic blood pressure reactivity to conflict.
Schmitz et al. [33]	RCT	Germany	53 BPD patients; 60 healthy controls (>18 yo)	Patients with BPD had significantly lower mean HEP amplitudes, which were negatively correlated with emotional dysregulation. Oxytocin had no effect on HEP amplitude.
Barber et al. [39]	cross-sectional study	United States	1295 subjects (30–54 yo)	BPD traits predicted cardiometabolic risk independently from depressive pathology.
Otto et al. [12]	case–control study	Germany	44 BPD female patients; 45 female healthy controls (mean age 25.9 ± 4.6 yo)	AL positively correlates with BPD. There was an indirect effect of early adversity on AL via PoLS.
Richter et al. [10]	longitudinal study	Germany	28 BPD–DDM patients; 22 DDM patients; 26 healthy controls (18–60 yo)	BPD is associated with an early, elevated risk of CVD (>EAT). Diabetes risk was significantly increased.
Engemann et al. [13]	case–control study	United States	50 BPD patients; 50 healthy controls (18–38 yo)	Altered GLS and increased AL in BPD. AL was significantly associated with GLS.
Kern et al. [42]	cross-sectional study	Germany	50 BPD patients; 49 healthy controls (>18 yo)	Higher Hcy and cardiovascular risk factors in BPD.
Abrahamian et al. [51] citing Kahl et al. [44]	position paper cross-sectional study	Austria	135 subjects (18–56 yo)	Metabolic syndrome was higher in subjects with BPD features.
Aweimer et al. [9]	case–control study	Germany	50 BPD patients; 50 healthy controls (18–38 yo)	GLS reduction in BPD. Increased cardiovascular risk factor in BPD.
Baptista et al. [15]	cross-sectional study	United States	892 BPD patients; 29,257 controls (>18 yo)	Higher BMI and metabolic parameters in BPD.

BPD: borderline personality disorder; BMI: body mass index; BP: bipolar disorder; GLS: global longitudinal strain; HEP: heartbeat-evoked potentials; hcy: homocysteine; CVD: cardiovascular disease; EAT: pericardiac adipose tissue; AL: allostatic load; PoLS: pace of life syndrome.
